# Molar Mass Thresholds in the Structural Behavior of Benzodithiophene-Based Semiconducting Polymers

**DOI:** 10.1021/acs.macromol.5c01743

**Published:** 2025-10-30

**Authors:** Matteo Sanviti, Jeromy Rech, Xiaowei Zhong, Wei You, Jaime Martín

**Affiliations:** † Universidade da Coruña Campus Industrial de Ferrol, CITENI, Campus de Esteiro S/N, Ferrol 15471, Spain; ‡ Department of Chemistry, 2331 University of North Carolina at Chapel Hill, Chapel Hill, North Carolina 27599-3290, United States

## Abstract

The performance of organic solar cells (OSCs) is tightly linked to the solid-state microstructure of their active layer components, particularly donor semiconducting polymers. Among these, benzodithiophene (BDT)-based polymers have gained attention due to their high power conversion efficiencies. In this study, we investigate how the molar mass of BDT-based polymers—specifically D18Cl and PBnDT-FTAZ—influences their general structural behavior (including the as cast solid-state microstructure, the thermotropic behavior, and their response to thermal annealing). Using techniques such as atomic force microscopy, grazing incidence wide-angle X-ray scattering, UV–vis spectroscopy, and fast scanning calorimetry, we show that ∼70 kg/mol is a threshold number-averaged molar mass, *M*_n_, value with respect to the solid-state microstructure of these materials. Specifically, ∼70 kg/mol polymers exhibit reduced domain size, a high degree of crystallinity (DoC), the strongest face-on orientation, a most blue-shifted absorption edge, and the highest mesophase melting temperature. Interestingly, the highest performing devices using these materials are fabricated with ∼70 kg/mol polymers, which suggests a direct connection between the molar mass of the donor polymer, its structural behavior, and device function. Furthermore, we reveal that segmental dynamics within the supercooled liquid phase govern the evolution of DoC during thermal annealing. Our findings underscore the importance of *M*_n_ tuning for optimizing the solid-state microstructure of BDT-based polymers and offer a refined framework for guiding the molecular design of high-efficiency OSCs.

## Graphical abstract

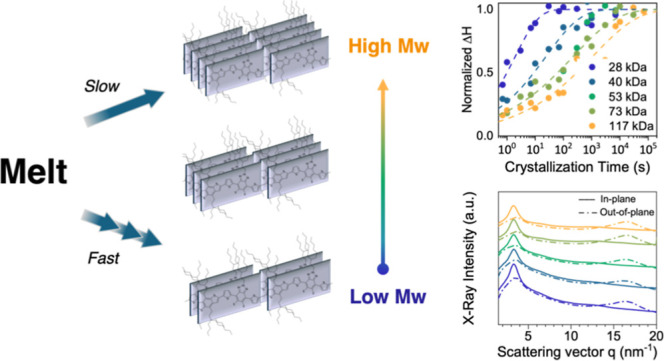

## Introduction

Semiconducting polymers gathered considerable interest in optoelectronics, significantly broadening their applications and enabling the rapid expansion of organic electronics field.^1,2^ It is well-recognized that optoelectronic properties of polymeric semiconductors are closely connected to their solid-state microstructure, i.e., to how polymer molecules are organized within the material.^3–7^ The solid-state microstructure of polymer semiconductors is mainly influenced by the processing conditions used for the device and material fabrication and by its own molecular characteristics. Among the latter, the molar mass stands out as a major aspect governing the solid-state microstructure of this class of materials.^8,9^ Consequently, understanding the impact of the molar mass on the microstructure of semiconducting polymers is of paramount importance for the rational optimization of organic electronic devices.^10^

For example, numerous investigations have established an interplay between the molar mass and the operation and performance of organic solar cells (OSCs).^10–22^ Overall, these works have shown that the use of donor benzodithiophene (BDT) polymers with optimal molar mass improves light absorbance,^10,15–17,19^ charge mobility,^17–19^ device stability,^14,19^ and thickness tolerance,^19^ along with ensuring better processability and mechanical properties.^20,21^ For example, moderately high molar mass values seem to be beneficial for FTAZ-based polymers. Li et al. reported maximum power conversion efficiency (PCE) values for devices containing a PBnDT-FTAZ polymer with a number-average molar mass (*M*_n_) of 60 kg/mol,^17^ while Liao et al. observed the maximum PCE for a *M*_n_ of 124 kg/mol PBnDTT-2Cl-FTAZ.^23^ Likewise, Zhong et al. reported that the optimal PCE value for D18Cl-based devices was achieved when using a 55 kg/mol polymer.^24^ Interestingly, Zeng et al. established that D18Cl devices only exhibit high PCE values when 60 < *M*_n_ < 70 kg/mol.^12^ Many of the reports addressed above have shown that the performance dependence on *M*_n_ is concomitant with microstructural variations in the BDT-based polymer, highlighting the need for a better understanding of the interplay of molar mass and the solid-state microstructure of high-performing BDT-based polymers used in OSCs.

At regular device operation temperatures spun cast films of BDT-based polymer tend to exhibit a biphasic solid-state microstructure that combines a solid mesophase structure that arranges in nanoscale fibril-like domains and glassy regions.^25^ The solid mesophase in BDT-based polymers seems to be a layered structure in which solid-like layers of π-stacked backbone segments and liquidlike layers of aliphatic side chains are alternated. Nevertheless, the fraction of mesophase domains (which could be related to the degree of crystallinity, DoC) can be high in polymers such as D18 or PM6 and glassy material regions can go unnoticed in these materials. Conversely, polymers such as PBnDT-FTAZ exhibit a detectable glassy phase and a glass transition temperature, *T*_g_.

In this work, we unveil the impact of molar mass on the structural behavior of the BDT-polymers. For that, our study selects D18Cl and PBnDT-FTAZ as model systems for BDT-based polymers exhibiting the two distinct structural behaviors observed in this class of polymers.^25^ Our study suggests that ∼70 kg/mol may be a critical *M*_n_ value in relation to the solid-state microstructure of these BDT-polymers, as ∼70 kg/mol polymers exhibit (i) the nanomorphology with reduced characteristic size; (ii) a high fraction of structurally ordered material, typically referred to as the degree of crystallinity (DoC); (iii) the strongest face-on molecular orientation; (iv) the most blue-shifted optical absorption edge; (v) and the highest *T*_m_ values (thus the mesophase structure with the higher thermodynamic stability). As mentioned above, maximum PCE values have been frequently reported for ∼70 kg/mol PBnDT-FTAZ^17^ and D18Cl,^24^ which might suggest a connection between the molar mass of the donor polymer, its structural behavior, and device function. Our study also concludes that segmental dynamics occurring in the supercooled liquid govern the advance of the DoC during thermal annealing of BDT polymers. This study provides a more refined framework for understanding how molar mass affects the solid-state macrostructure of high-performing polymers for organic solar cells, contributing to a better understanding of these important materials.

## Results and Discussion

PBnDT-FTAZ polymers with a number-average molar mass (*M*_n_) of 28 kg/mol (*D̵* = 2.0), 40 kg/mol (*D̵* = 1.9), 53 kg/mol (*D̵* = 2.3), 73 kg/mol (*D̵* = 2.5), and 117 kg/mol (*D̵* = 2.2); D18Cl polymers with a *M*_n_ of 30 kg/mol (dispersity, *D̵* = 4.8), 46 kg/mol (*D̵* = 5.4), 77 kg/mol (*D̵* = 2.6) and 89 kg/mol (*D̵* = 5.0) kg/mol were studied. The molecular structure of the studied polymers is depicted in Scheme 1. Synthetic procedures for D18Cl and PBnDT-FTAZ are reported in refs 22, 24, and 26 , respectively. High-temperature gel permeation chromatography (HT-GPC) traces of different batches of FTAZ and D18-Cl are included in Figures S1 and S2 of the Supporting Information. It should be noted that the *M*_n_ values referred above are calculated by size exclusion chromatography based on polystyrene standards, which is known to overestimate *M*_n_ values by a factor that escalates with the *M*_n_.^27^ The polymers listed above were dissolved in chlorobenzene (10–15 mg/mL solutions) and spun cast into thin films.

**Scheme 1:**
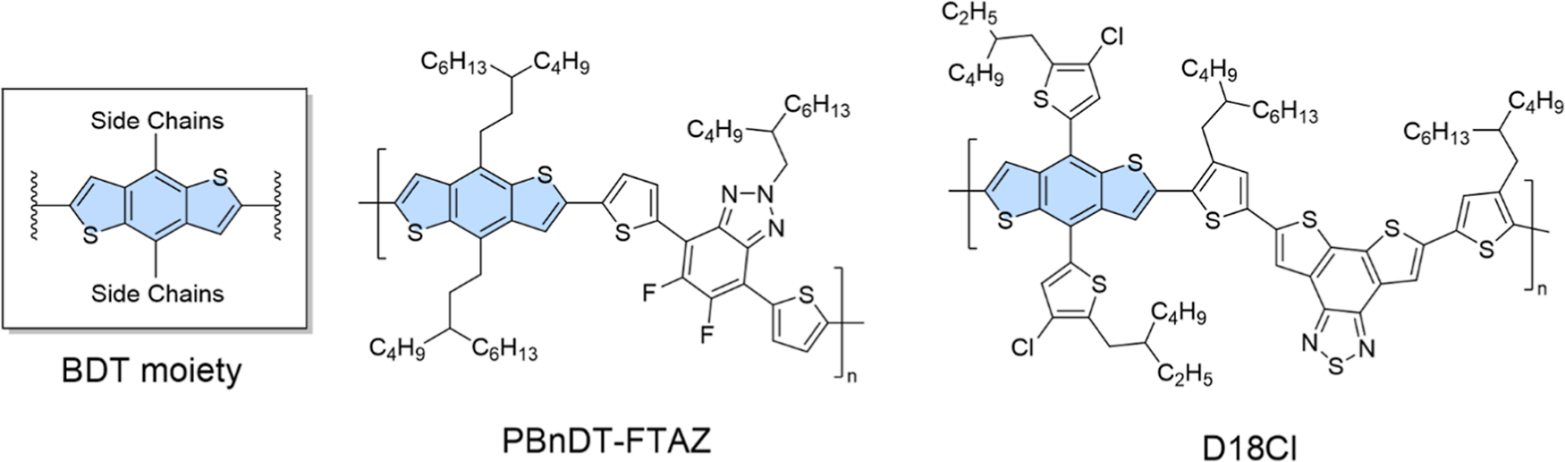
Chemical Structures of the Studied Polymer Compounds

We first assessed the impact of *M*_n_ on the solid-state microstructure and morphology of spun cast D18Cl and PBnDT-FTAZ thin films. In agreement with previous results,^25,28,29^ D18Cl films showed a nanofibrillar morphology when inspected by atomic force microscopy (AFM), while PBnDT-FTAZ samples contained granular-like features. Shown in Figure 1a,b are topography images for the 77 kg/mol D18Cl and the 73 kg/mol PBnDT-FTAZ samples, respectively. Analogous images for the rest of the samples are included in Figures S3 and S4 of the Supporting Information. Table 1 summarizes the morphological parameters extracted from AFM analysis. Our analysis reveals that as the *M*_n_ of D18Cl increases, the surface roughness, *R*_a_, rises (from 0.33 to 0.41 nm as the *M*_n_ is increased from 30 to 89 kg/mol). Moreover, the analysis of the spatial frequencies (ν_max_) of power spectral density functions (PSDF) of AFM images reveals that the characteristic size of the nanofibrillar morphology (represented by the value of 1/ν_max_) reduces on increasing the *M*_n_ (e.g., from 29.8 nm of the 30 kg/mol sample to 24.1 nm of the 89 kg/mol sample; see Table 1). PBnDT-FTAZ samples exhibited lower *R*_a_ values (Table 1) with little dependence on the *M*_n_. Interestingly, however, here again, the smallest characteristic size of the morphology is found for the high-*M*_n_ samples, the 73 kg/mol sample being the one with the smallest characteristic length scale (Table 1). We note that, in general, high PCE values benefit from reduced structural features (e.g., crystallites, separation between crystals), as these are required for the formation of a suitable bulk heterojunction nanomorphology. For example, large crystals result in excessive donor/acceptor phase separation and excessive exciton recombination. Small, nanoscale crystallites, however, should not distort significantly the donor/acceptor nanomorphology yielding good photovoltaic performance.

**Figure 1:**
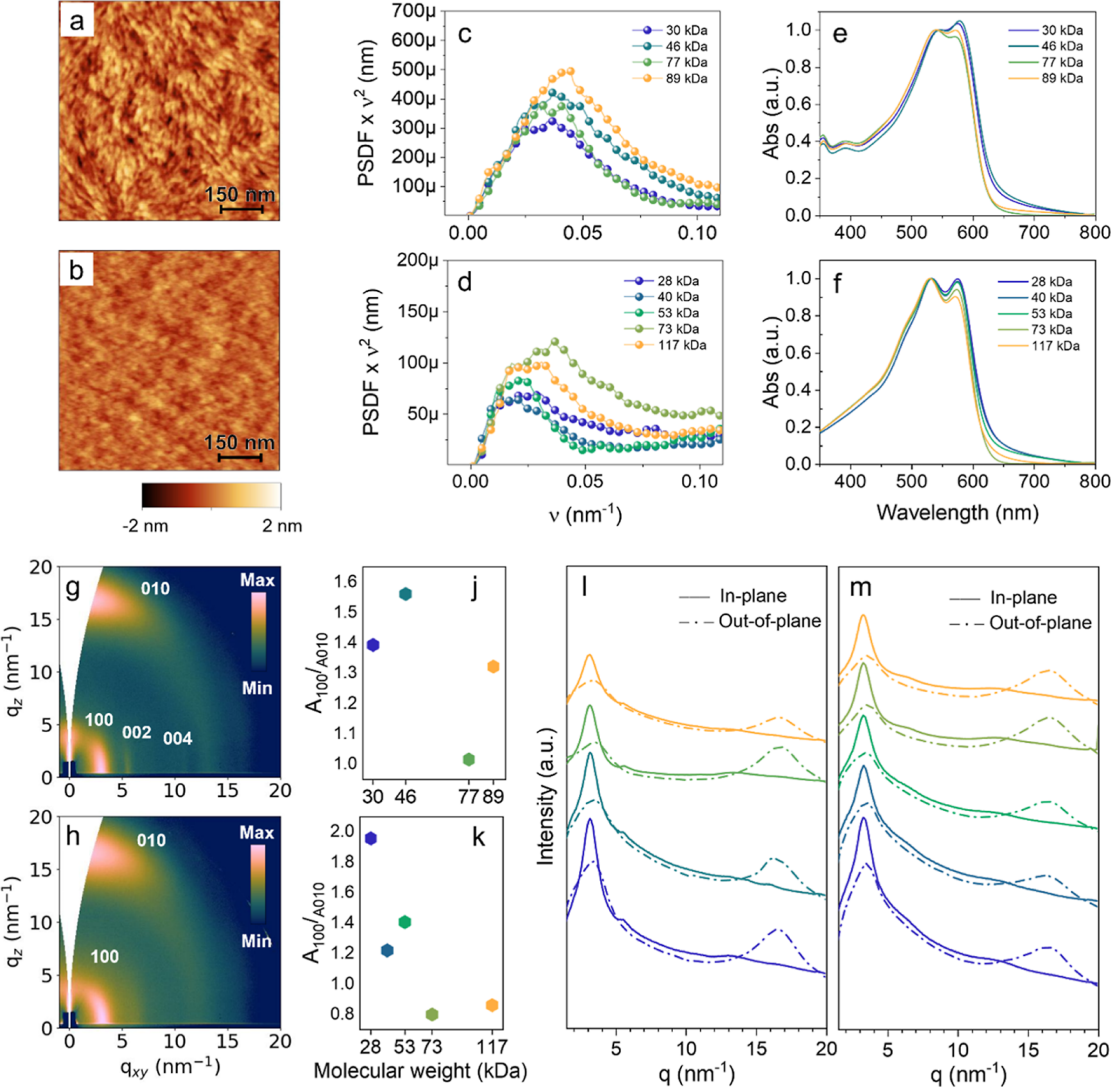
750 × 750 nm AFM topography images of 77 kg/mol D18Cl (a) and 73 kg/mol PBnDT-FTAZ (b) and relative PSDF (c,d), full data set in Figures S3 and S4. (e,f) UV–vis absorption spectra acquired on dried thin films. Grazing incidence wide-angle X-ray scattering (GIWAXS) diffraction images of 77 kg/mol D18Cl (g) and 73 kg/mol PBnDT-FTAZ (h). (l,m) 1D integration cuts from GIWAXS 2D images, included in Figure S5 (normalized to the film thickness). (j,k) Ratio of the areas of the peaks 100 and 010 as a function of molar mass calculated from the 1D integration cuts.

**Table 1:** Values of Intensity, *q*_peak_, and CCL of the 100 and 010 Diffraction Peaks from GIWAXS Images in Figure S5, Topography Roughness and Spacing Period Calculated from the AFM Images, Wavelength of the 0–0 and 0–1 Peaks Maximum from UV–Vis Spectroscopy

		100 plane				010 plane						
sample	*M*_n_ (kg/mol)	Int. (au)	*q* (nm^–1^)	CCL (nm)	*g* (%)	Int. (au)	*q* (nm^–1^)	CCL (nm)	*g* (%)	*R*_a_ (nm)	1/ν_max_ (nm)	*I*_0–0_/*I*_0–1_
D18Cl	30	1410	3.27	3.2	30	425	16.61	1.7	18	0.33	29.8	1.03
	46	851	3.27	3.2	29	289	16.53	1.8	18	0.37	25.9	1.05
	77	1910	3.21	3.2	29	871	16.60	1.7	18	0.33	27.5	0.96
	89	1045	3.25	3.0	30	361	16.67	1.7	18	0.41	24.1	1.00
PBnDT-FTAZ	28	989	3.42	3.5	27	219	16.48	1.8	17	0.18	40.1	1.00
	40	1400	3.42	3.6	27	427	16.55	1.8	18	0.21	54.1	0.99
	53	924	3.37	3.5	28	263	16.48	1.7	18	0.19	46.8	0.98
	73	2120	3.35	3.6	27	948	16.43	1.5	19	0.21	28.6	0.94
	117	1770	3.34	3.5	28	721	16.47	1.6	19	0.21	34.2	0.90

Representative grazing incidence X-ray scattering 2D images of the 77 kg/mol D18Cl sample and the 73 kg/mol PBnDT-FTAZ samples are shown in Figure 1g,h, respectively. Counterpart GIWAXS data of the rest of the samples are included in Figure S5 of the Supporting Information. All D18Cl samples display diffraction peaks at scattering vector, *q*, values of ∼3.2 nm^–1^ (mainly along the in-plane directions) and ∼16.6 nm^–1^ (along the out-of-plane direction) that can be ascribed to the (100) and (010) planes, respectively. Furthermore, a narrow peak from the (00*l*) planes shows up at *q* = ∼5.5 nm^–1^ in the in-plane. Peaks from the (100) and (010) planes in PBnDT-FTAZ samples are centered at *q* = ∼3.4 nm^–1^ (in-plane) and ∼16.5 nm^–1^ (out-of-plane), respectively (Figures 1h and S5 of the Supporting Information). Thus, independently of the *M*_n_, both D18Cl and PBnDT-FTAZ exhibit a preferential face-on orientation. Moreover, the degree of face-on orientation, parametrized in the ratio of intensities of the (100) and (010) peaks, *A*_100_/*A*_010_, seems to decrease as the *M*_n_ is increased (Figure 1j,k). Interestingly, for both D18Cl and PBnDT-FTAZ, the maximum degree of face-on orientation is measured on samples with *M*_n_ around 70 kg/mol (i.e., on 77 kg/mol for D18Cl and 73 kg/mol for PBnDT-FTAZ).

Radial integrations of the GIWAXS intensity are shown in Figure 1l,m. The overall intensity of diffraction peaks seems to increase as the *M*_n_ of both materials increases, especially for PBnDT-FTAZ, which suggests that the DoC may be greater in these materials. This enhancement of the DoC seems to be linked to both a blue-shift of the UV–vis absorption edge, which is found to be maximum for the 77 kg/mol D18Cl and 73 kg/mol PBnDT-FTAZ samples, as well as with a reduction of the ratio of intensities between the 0–0 and the 0–1 transition bands, *I*_0–0_/*I*_0–1_ (see Figure 1e,f). GIWAXS, peak analysis suggests that the *M*_n_ does not have a strong impact on the overall quality of structural order, as neither the X-ray coherence length (CCL) nor the lattice distortion parameter, *g*-parameter, change significantly among samples (Table 1). CCL values are calculated as $=\frac{2\pi k}{\Delta q}$ , where *q* is the scattering vector at the maximum intensity of the peak of interest, Δ*q* is the full width at half-maximum of the peak, and *k* is a constant fixed to 0.9. *g*-parameters are obtained from first-order GIWAXS peaks as $g\approx \frac{1}{2\pi }\sqrt{\Delta q\cdot d_{hkl}}$ where *d*_*hkl*_ is the *d*-spacing of the diffraction peak.^30^

Having obtained a detailed picture of the impact of *M*_n_ on the solid-state microstructure/morphology, we then investigated the interplay between *M*_n_ and the thermal behavior of D18Cl and PBnDT-FTAZ in relation to structure. The impact of *M*_n_ on the melting and “crystallization” temperatures of the mesophase, denoted as *T*_m_ and *T*_c_, respectively, was investigated by fast scanning calorimetry (FSC) experiments. Shown in Figure 2a,b are the heat flow rate (HF) vs temperature traces, conducted at 2000 °C/s, for D18Cl and PBnDT-FTAZ polymers, respectively. Moreover, in order to increase the intensity of melting peaks (e.g., those of low-*M*_n_ PBnDT-FTAZ polymers), a further set of samples were molten at 450 °C and then “crystallized” at 260 °C for 30 min. FSC heating traces of the thus prepared samples are shown in Figure 2c,d. We note that to further increase the clarity of these results, baselines were corrected by subtracting the heating traces of same samples quenched at −4000 °C/s from the melt state. Subsequent cooling scans from the melt (at −1000 °C) are shown in Figure 2e,f. The *T*_m_ and *T*_c_ values obtained from these experiments, as well as the supercooling required for crystallization, i.e., Δ*T* = *T*_m_ – *T*_c_, are summarized in Table 2. Tellingly, for both D18Cl and PBnDT-FTAZ the maximum *T*_m_ correspond to polymers with *M*_n_ of ∼70 kg/mol (more precisely, 77 kg/mol for D18Cl and 73 kg/mol for PBnDT-FTAZ), which suggests that these *M*_n_ values yield mesophase structures of larger thermodynamic stability. A similar nonmonotonic trend was previously observed in poly­(3-hexylthiophene) (P3HT).^8^ It was rationalized in terms of a transition from extended-chain crystals to chain-folded crystals, as the *T*_m_ is typically governed by the lamellar thickness in typical polymer crystals. Because PBnDT-FTAZ and D18Cl do not form traditional lamellar crystals such as semicrystalline polymers, this argument seems at least partially invalid in this case. Yet, it cannot be ruled out that polymers with *M*_n_ of ∼70 kg/mol arrange into mesophase structures with the longest size along the extended-chain direction (i.e., the crystallographic [001] direction). Similarly, the highest *T*_c_ and Δ*T* values are found in polymers with *M*_n_ of ∼70 kg/mol. It is also worth noting that D18Cl requires much larger supercoolings (ranging from 59 to 88 °C) than PBnDT-FTAZ (from 10 to 23 °C).

**Figure 2:**
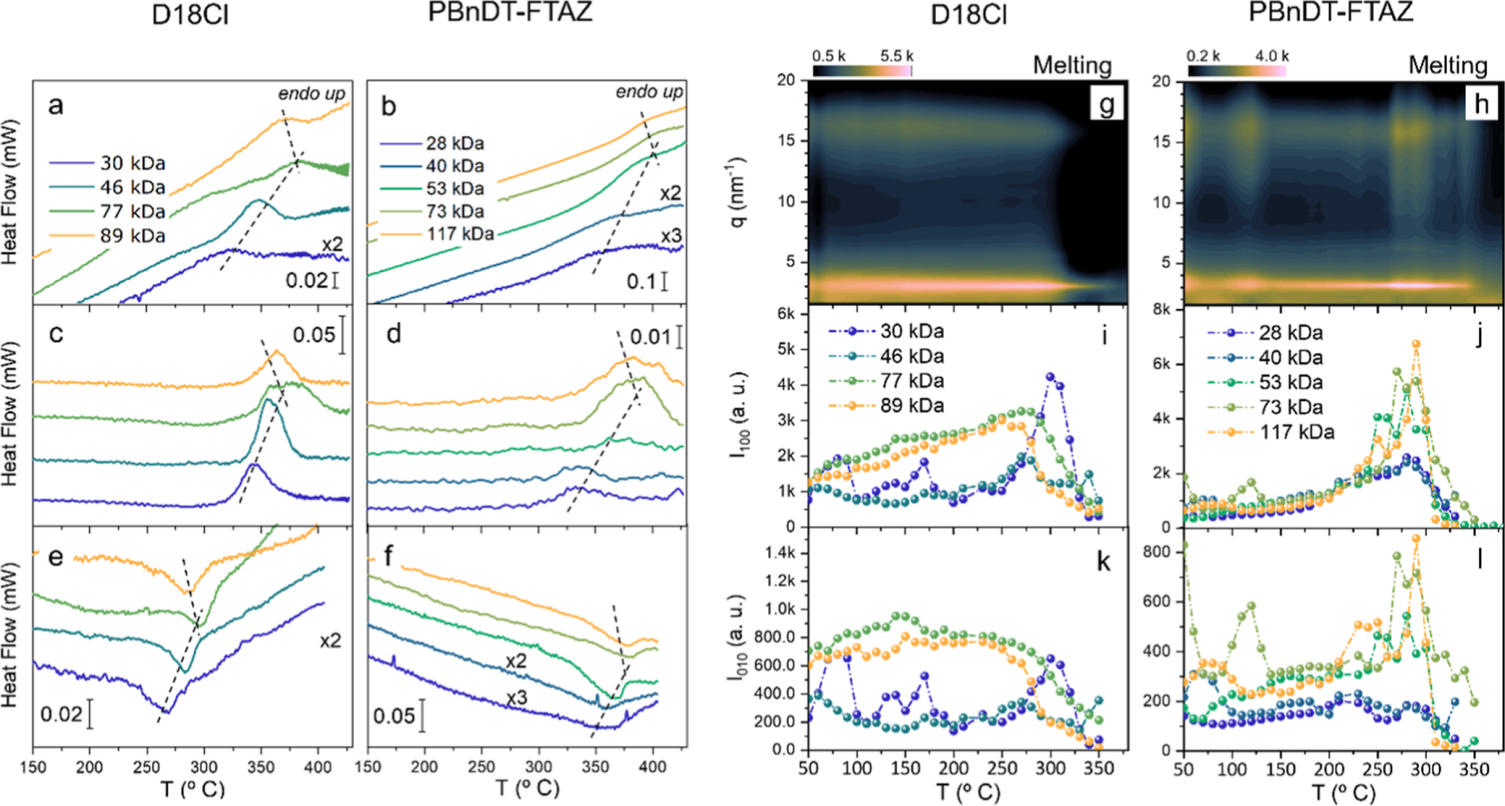
FSC second heating (a,b) and cooling (e,f) traces in flash-DSC and the ΔHF after annealing at 260 °C (c,d). (g,h) 1D integration of the GIWAXS patterns obtained from the in situ experiment on D18Cl (77 kg/mol) (g) and PBnDT-FTAZ (73 kg/mol) (h). (i–l) Intensity of (100) (i,j) and (010) (k,l) peaks as a function of temperature recorded during a heating ramp at 15 °C/min.

**Table 2:** Melting Temperature (*T*_m_), Crystallization Temperature (*T*_c_), and Supercooling (Δ*T* = *T*_m_ – *T*_c_) Calculated from Curves Shown in Figure 2 ^a^

sample	*M*_n_ (kg/mol)	*T*_m_ (°C)	*T*_c_ (°C)	*T*_m260_ (°C)	Δ*T* (°C)
D18Cl	30	327	268	344	59
	46	350	285	357	65
	77	386	298	376	88
	89	375	287	364	88
PBnDT-FTAZ	28	358	348	336	10
	40	372	358	336	14
	53	390	368	373	22
	73	404	381	388	23
	117	398	379	383	19

a *T*_m260_ is the melting temperature of crystals formed during annealing at 260 °C.

In situ temperature-resolved GIWAXS data recorded during heating for 77 kg/mol D18Cl and 73 kg/mol PBnDT-FTAZ samples (at 15 °C/min) are shown in Figure 2g,h, respectively. Here, the integrated GIWAXS intensity is plotted on a color scale vs *q* and temperature. Analogous data for the rest of the polymers are included in Figure S6 of the Supporting Information. To best visualize the structural changes occurring during heating, the intensities of the (100) and (010) peaks for all D18Cl and PBnDT-FTAZ samples are plotted vs temperature in Figure 2i–l. We note, moreover, further relevant structural parameters, namely, the CCL, the *g*-parameter, and the *q*-value, are included in Figure S7. Structural parameters obtained from the GIWAXS diffractograms depicted in Figure S6. Interestingly, both polymers exhibit markedly different behaviors. High *M*_n_ D18Cl polymers show a constant increase of the (100) peak intensity during heating, while the (010) peak remains rather constant, suggesting a mild overall impact of thermal annealing on the structure. Conversely, the highest-*M*_n_ PBnDT-FTAZ materials exhibit a rapid increase of the structural order, along both the [100] and the [010] crystallographic directions, at temperatures above 200 °C. This is likely connected with the different DoC values of both materials. It is also worth noting that the lowest-*M*_n_ PBnDT-FTAZ polymers seem to be rather insensitive to temperature, which may result from the low tendency to crystallize low-*M*_n_ PBnDT-FTAZ polymers.

Motivated by these findings, we studied the impact of *M*_n_ on the structural development of BDT-based polymers during isothermal annealing. For that, we began analyzing the impact of *M*_n_ on their thermotropic phase behavior. Materials were first investigated by FSC, using a thermal protocol shown in Figure S8a and reported elsewhere.^31–33^ Briefly, the materials were subjected to a temperature above the melting temperature of the mesophase to erase the thermal history (e.g., 420 °C). Subsequently, the samples were cooled at −4000 °C/s to a specific isothermal temperature (*T*_a_) and kept there for 30 min. *T*_a_s ranged from −80 to 280–300 °C. During the annealing at *T*_a_ the materials will undergo evolution in accordance with the dictates of its thermodynamic properties. For example, if *T*_a_ falls within the temperature range in which the material is a glass, it will undergo physical aging. Thus, a subsequent heating scan will display an endothermic overshoot at temperatures close to *T*_g_, reflecting the recovery of the enthalpy level of the supercooled liquid phase. Conversely, if *T*_a_ is within the temperature range in which the material contains a supercooled liquid state, the system will attempt to crystallize via cold crystallization, and likewise, it will show an endothermic crystal melting peak in the subsequent heating scan. Figure 3a,b shows the heating scans of the isothermally annealed 77 kg/mol D18Cl sample and the 73 kg/mol PBnDT-FTAZ samples, respectively (sample-scans in Figure Sa8), compared to the heating scans of the same nonannealed samples (reference-scans in Figure S8a). Clearly, isothermal steps result in the emergence of endothermic peaks that increase and decrease in intensity depending on the applied *T*_a_, and thus, they can be used to screen the thermotropic landscape of materials. Similar data for the rest of *M*_n_s are included in Figures S9–S10 of the Supporting Information. Furthermore, shown in Figure 3c,d are the enthalpy changes (Δ*H*, in arbitrary units) associated with the structural evolution of all materials during the isothermal steps against the *T*_a_ applied. This plot provides a clearer view of the structurally relevant temperature ranges and the phase transition temperatures. We note that the *T*_a_s at which the Δ*H* is equal to zero correspond to the upper limit of the phase transition temperature.

**Figure 3:**
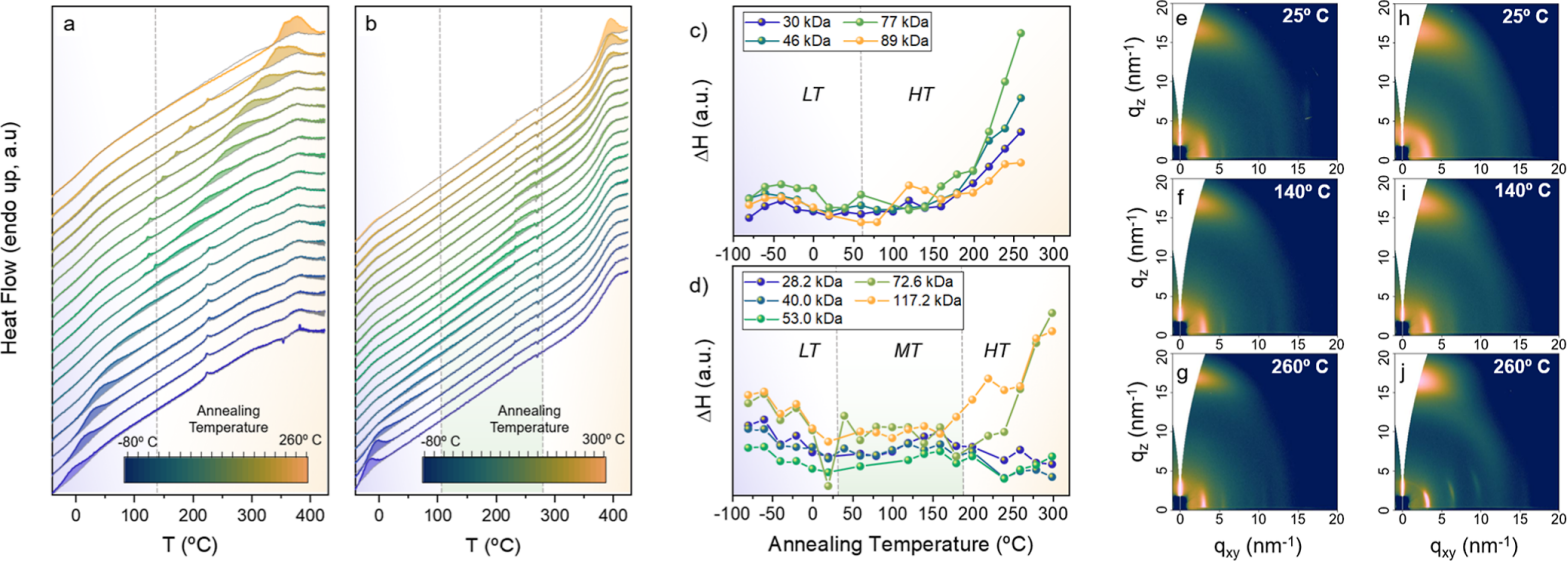
Results from flash-DSC experiment in terms of heat flow (a,b) and Δ*H* (c,d) for 77 kg/mol D18Cl. Plots a and b, respectively, refer to 77 kg/mol D18Cl and 73 kg/mol PBnDT-FTAZ. On the right, GIWAXS patterns of 77 kg/mol D18Cl (e–g) and 73 kg/mol PBnDT-FTAZ (h–j) as cast thin films (e,h) and annealed for 10 min at 140 °C (f,i) and 260 °C (g,j).

In good agreement with the conclusions of our previous report,^25^ D18Cl displays two temperature regions with different thermal response in the solid state, i.e., a so-called high-temperature (HT) state and a low-temperature state. This phase behavior is moreover independent of the *M*_n_. In contrast, PBnDT-FTAZ seems to exhibit three distinct temperature regions, including a medium temperature (MT) state in addition to those mentioned above. Nevertheless, the HT state in PBnDT-FTAZ is more noticeable in high-*M*_n_ polymers as lower *M*_n_ PBnDT-FTAZ samples exhibit a reduced tendency to crystallize. As reported elsewhere, the HT state is characterized by the coexistence of a solid mesophase along with other regions where the material is in a supercooled liquid phase. In the MT state, however, disordered regions are vitrified.

Figure 3c,d also reveals that during thermal annealing at temperatures within the HT state, the Δ*H* of melting of D18Cl and high-*M*_n_ PBnDT-FTAZ polymers increases, which indicates the rise of the DoC. Interestingly, here again, the ∼70 kg/mol polymers stand out as the materials featuring the fastest DoC growth (in good accordance with the increase of the GIWAXS intensity shown in Figure 2). The increase in the DoC due to thermal annealing is found to be minor in low-*M*_n_ PBnDT-FTAZ, likely due to the inherently low tendency of these polymers to crystallize.

Once the thermotropic landscape of materials was assessed, we analyzed the *M*_n_-effect on the structural development of materials during isothermal annealing at selected temperatures, i.e., 140 and 260 °C. Figure 3e–g compares GIWAXS patterns of 77 kg/mol D18Cl films annealed at room temperature, at 140 °C, and at 260 °C, respectively. Figure 3h–j display analogous data for the 73 kg/mol PBnDT-FTAZ, while data for the rest of polymers are included in Figures S11 and S12 of the Supporting Information. Moreover, the structural parameters extracted from the GIWAXS analysis are summarized in Table 3. Although thermal annealing at 140 °C induces some mild structural changes, e.g., a weak improvement of the lamellar periodicity both in D18Cl and PBnDT-FTAZ polymers, the major changes occur when the materials are annealed at 260 °C, i.e., at temperatures within the HT region. Consequently, we focus our discussion on samples annealed at 260 °C.

**Table 3:** Structural Parameters Obtained from GIWAXS Diffractograms Peak Fitting Shown in Figures S11 and 12

		CCL (nm)			*g* (%)		
annealing temperature →	sample (Mn)	as cast	140 °C	260 °C	as cast	140 °C	260 °C
D18Cl plane 100	30 kg/mol	6.1	7.2	13.7	27	21	15
	46 kg/mol	5.8	9.3	13.5	30	18	15
	77 kg/mol	5.8	6.6	10.1	30	24	17
	89 kg/mol	5.7	6.8	10.6	32	23	16
D18Cl plane 010	30 kg/mol	1.3	1.3	1.3	18	17	15
	46 kg/mol	1.2	1.1	1.3	18	16	15
	77 kg/mol	1.1	1.2	1.3	19	17	16
	89 kg/mol	1.1	1.2	1.3	19	19	17
FTAZ plane 100	28 kg/mol	5.6	8.9	23.7	25	17	13
	40 kg/mol	5.6	8.1	23.0	25	20	13
	53 kg/mol	5.5	7.5	22.1	28	19	11
	73 kg/mol	5.4	7.0	19.3	28	20	11
	117 kg/mol	5.4	7.1	18.5	26	21	12
FTAZ plane 010	28 kg/mol	1.2	1.1	1.3	21	18	16
	40 kg/mol	1.1	1.1	1.1	20	18	20
	53 kg/mol	1.1	1.1	1.1	20	18	17
	73 kg/mol	1.1	1.1	1.1	20	18	17
	117 kDa	1.1	1.1	1.1	20	18	17

Due to the improvement of structural order during annealing, the CCL values for the (100) stack of planes of all polymers annealed at 260 °C are significantly higher than those of as cast polymers (see Table 3). Interestingly, this CCL enhancement is found to be dependent on the *M*_n_ for both D18Cl and PBnDT-FTAZ: the larger the *M*_n_, the smaller the increase of the CCL. For example, while the CCL of the 28 kg/mol PBnDT-FTAZ increases by a factor of 4.2, the CCL increases 3.5 times for the 117 kg/mol FTAZ. Likewise, the CCL of the 30 kg/mol D18Cl increases 2.2 times after being annealed at 260 °C, while an increase of 1.8 times is found for the 89 kg/mol D18Cl. Similarly, the *g*-parameters measured for the (100) planes decrease significantly (by a factor of 2, approximately) upon annealing both PBnDT-FTAZ and D18Cl polymers at 260 °C. Interestingly, while the decrease of the *g*-parameter in D18Cl shows no significant *M*_n_-effect, the decrease of the *g*-parameters in PBnDT-FTAZ polymers is more profound as the *M*_n_ decreases (see Figure S7).

In order to further understand the structural changes occurring during thermal annealing, we investigated the kinetics of structural development at 260 °C by FSC. For that, we analyzed the increase of enthalpy after annealing samples at 260 °C for different times (ranging from 10^–1^ to 10^5^ s) applying the thermal protocol shown in Figure S8b of the Supporting Information. Figure 4a,b shows the HF vs *T* curves for the 77 kg/mol D18Cl and the 73 kg/mol PBnDT-FTAZ, corresponding to the heating scans sample- and reference-shown in Figure S8b of the Supporting Information. The data obtained for the rest of polymers are shown in Figure S13 of the Supporting Information. The integration of the ΔHF curves (shown in Figure S13 of the Supporting Information) provides the change of enthalpy (Δ*H*) resulting from each annealing period applied. The evolution of the Δ*H* with time is shown in Figure 4c,d for all polymers, where solid symbols represent the experimentally measured Δ*H* (normalized to the final Δ*H* value, ${\Delta H}_{t\to \infty }$ ) and dashed lines are fits to the Avrami law: $\frac{\Delta H}{{\Delta H}_{t\to \infty }}=e^{-k{\left(t-t_{0}\right)}^{n}}$ , where *K* is an overall rate constant and *n* is the so-called Avrami exponent.^34^ The calculated *K* and *n* values are included in Table 4. Except for the 28 kg/mol PBnDT-FTAZ, all polymers exhibit as *n* of ∼0.3, thus far from the typical Avrami exponents found in the crystallization of flexible polymers.^35^ However, this is a typical value for stretching exponents of glassy dynamics.^31^ Consequently, we argue that segmental dynamics occurring in the supercooled liquid of the HT state govern the kinetics of structural changes during thermal annealing at 260 °C. This can also explain the decrease of the rate of structural changes, parametrized by the rate constant *K*, with the *M*_n_ observed for both materials. Moreover, we also observed that the *K* value of PBnDT-FTAZ depends more strongly on the *M*_n_ value than that of D18Cl. For example, while *K* ranges between 0.53 s^–*n*^ (for 28 kg/mol) and 0.22 s^–*n*^ (for 89 kg/mol) in D18Cl, their counterpart PBnDT-FTAZ values expand from 0.70 s^–*n*^ (for 30 kg/mol) to 0.15 s^–*n*^ (for 117 kg/mol). This observation is also in accordance with the larger dependence of CCL and the *g*-parameter on the *M*_n_ found in PBnDT-FTAZ compared to D18Cl, as discussed above (see also Figure S7).

**Figure 4:**
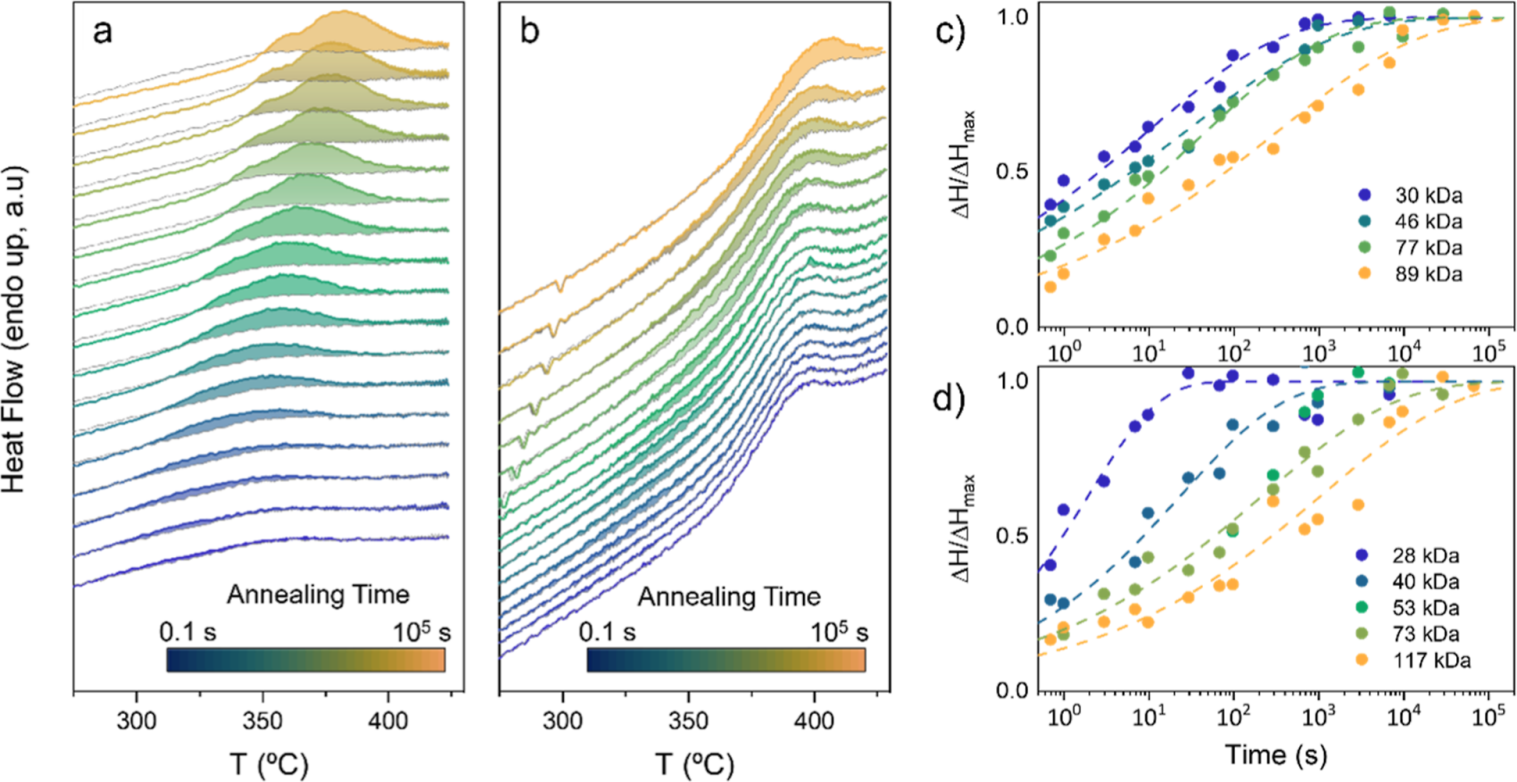
On the left, thermograms of D18Cl 77 kg/mol (a) and PBnDT-FTAZ 73 kg/mol (b) for different times of isothermal annealing at 260 °C; on the plots, the area corresponding to the characteristic Δ*H* is highlighted following the color of the relative line. On the right, the crystallization kinetics at 260 °C in all samples of D18Cl (c) and PBnDT-FTAZ (d). Enthalpy data were obtained from the integral area of ΔHF calculated from the curves shown in Figure S13.

**Table 4:** Kinetic Parameters Obtained from Fitting the Δ*H* Values to the Avrami Law

D18Cl			PBnDT-FTAZ		
*M*_n_ (kg/mol)	*K* (s^–*n*^)	*n*	*M*_n_ (kg/mol)	*k*	*n*
28	0.53	0.28	28	0.7	0.52
46	0.44	0.25	40	0.32	0.36
77	0.31	0.30	53		
89	0.22	0.26	73	0.22	0.28
			117	0.15	0.27

## Conclusions

In summary, the impact of the *M*_n_ on the structural behavior of the model-system BDT-polymers PBnDT-FTAZ and D18Cl is investigated by a combination of FSC, AFM, and GIWAXS. The main conclusion of our study is that ∼70 kg/mol seems to be a critical *M*_n_ value in relation to the microstructure of these BDT-polymers. The main rationales for concluding this are as cast films of polymers with a *M*_n_ = ∼70 kg/mol exhibit (i) a nanomorphology with reduced characteristic length scale; (ii) high DoC (this is especially noticeable in FTAZ, where low-*M*_n_ have a little tendency to crystallize); (iii) the acutest face-on molecular orientation; (iv) the most blue-shifted UV–vis absorption bands; (v) and the highest *T*_m_ values. Importantly, ∼70 kg/mol PBnDT-FTAZ and D18Cl are frequently found to exhibit maximum PCE values in organic solar cells,^17^ which suggests a correlation between—at least some of—the structural parameters mentioned above and device performance. Moreover, we conclude that segmental dynamics occurring in the supercooled liquid govern the advance of the DoC during thermal annealing of BDT polymers, as a time dependence of *t* ^∼0.3^ is observed. We anticipate that the new correlations revealed in our work between molar mass and solid-state microstructure in semiconducting polymers will contribute to a better understanding of these complex materials and ultimately to improve molecular design-function models for rational device engineering.

## Materials and Methods

### Materials

The D18Cl was synthesized as reported in ref 24. PBnDT-FTAZ was synthesized as reported in refs 22 and 26 . To characterize the molar mass of samples, HT-GPC measurements were performed on an Agilent 1260 HT-GPC instrument with TCB (1,2,4-trichlorobenzene) as the eluent at 150 °C. The obtained molar mass is relative to the polystyrene standard.

### Sample Preparation

The thin films were prepared by spin coating chlorobenzene solutions with polymer concentrations between 10 and 15 mg/mL. Si substrate was employed for GIWAXS and AFM measurements, while for the UV–vis analysis, we used glass slides. Film thicknesses, measured by an Alpha-Step D-600 profiler, ranged from 50 to 200 nm. The different film thicknesses are a consequence of the different molar masses of the polymers and the fact that similar solution concentrations were used for the preparation of all of the samples. The thermal treatments to samples for UV–vis and GIWAXS analysis was performed by using a Linkam hot stage under N_2_ atmosphere. The annealing time for each selected temperature was fixed to 10 min.

### Characterization

#### Grazing Incidence Wide-Angle X-ray Scattering

The experiments were performed at the BL11 NCDSWEET beamline at the ALBA Synchrotron Radiation Facility (Spain). Wide-angle X-ray scattering measurements were performed in grazing-incidence geometry, using a beam of 50 μm in width, energy of 12.4 keV, and 1 nm wavelength. Image acquisition was carried out through a 1 s exposure to a Rayonix LX255-HS detector put at 21 cm of distance. The α_i_ beam incidence angle was fixed to 0.12°, which was found to be the best compromise for an effective crystallographic planes imaging. For the setup calibration we made use of a Cr_2_O_3_ sample; the data were then expressed in terms of the scattering vector *q*, having dimensions nm^–1^. For the in situ temperature modulation experiments, a Linkams THMS 600 stage adapted for grazing incidence experiments was used, with heating rate of 15 °C/min and under an N_2_ atmosphere. Sample alignment was performed before each image acquisition.

#### Fast Scanning Calorimetry

FSC measurements were carried out using a Mettler Toledo Flash DSC+1, equipped with a two-stage intracooler under a nitrogen purge (75 mL min^–1^ N_2_ flow). Prior to use, the MultiSTAR UFS1MEMS chip sensors were conditioned and calibrated according to standard procedures. For sample preparation, polymer solutions were spin-coated onto the backside of the chip sensors. The second heating scan was conducted from −90 to 450 °C at a rapid heating rate of 4000 °C s^–1^. Before this, the films were cooled from 450 °C back to −90 °C at the same rate. Detailed information on isothermal annealing experiments is included in the text.

#### UV–Vis Spectroscopy

UV–vis spectra were recorded with a Shimadzu UV-2550 spectrometer with a film adapter.

#### Atomic Force Microscopy

Quantitative information about the film nanomorphology was obtained from power spectral density analysis applied to the AFM phase-contrast images using the Gwyddion software (version: 2.64.20231122). AFM data were collected in a CSI Instruments Nano-Observer AFM microscope operating with Nano Solution software (version: 1.37.0.405-SA1). The images were acquired using intermitted contact (resonant oscillating) mode, with amplitude set point fixed at 1.4 V. The tip employed for the measurements was the APP Nano ACT (tip of <10 nm radius, resonant frequency of 286.4 kHz, and spring constant between 25 and 45 N/m). The cantilever presented the following geometrical aspect: length ≈125 μm, width ≈35 μm, thickness ≈4 μm, and a cone length of about 15 μm.

## Supplementary Material



## Notes

HT-GPC traces of different batches, AFM topography images, 2D GIWAXS images, 1D integration of the GIWAXS patterns obtained from temperature-resolved in situ experiments, structural parameters as a function of temperature, thermal protocols and data of the FSC experiments, 2D GIWAXS data obtained after annealing at different conditions, and isothermal FSC results (PDF)
