# Supplementary material for: Molar Mass Thresholds in the Structural Behavior of Benzodithiophene-Based Semiconducting Polymers
Source: Macromolecules. 2025 Oct 30;58(21):11991–9. doi: 10.1021/acs.macromol.5c01743 (PMC12613820; doi:10.1021/acs.macromol.5c01743)
Supplement: Supplementary file 1 [file ma5c01743_si_001.pdf]

# Supplementary information for

# Molar Mass Thresholds in the Structural Behavior of Benzodithiophene-Based Semiconducting Polymers

Matteo Sanviti[1], Jeromy Reich [2], Xiaowei Zhong[2], Wei You[2], Jaime Martín*[1.]

[1.] Universidade da Coruña, Campus Industrial de Ferrol, CITENI, Campus de Esteiro S/N, 15471 Ferrol, Spain.

[2.] Department of Chemistry, University of North Carolina at Chapel Hill, Chapel Hill, North Carolina 27599-3290, United States.

**Corresponding author:**

**Jaime Martín** - Email: jaime.martin.perez@udc.es .

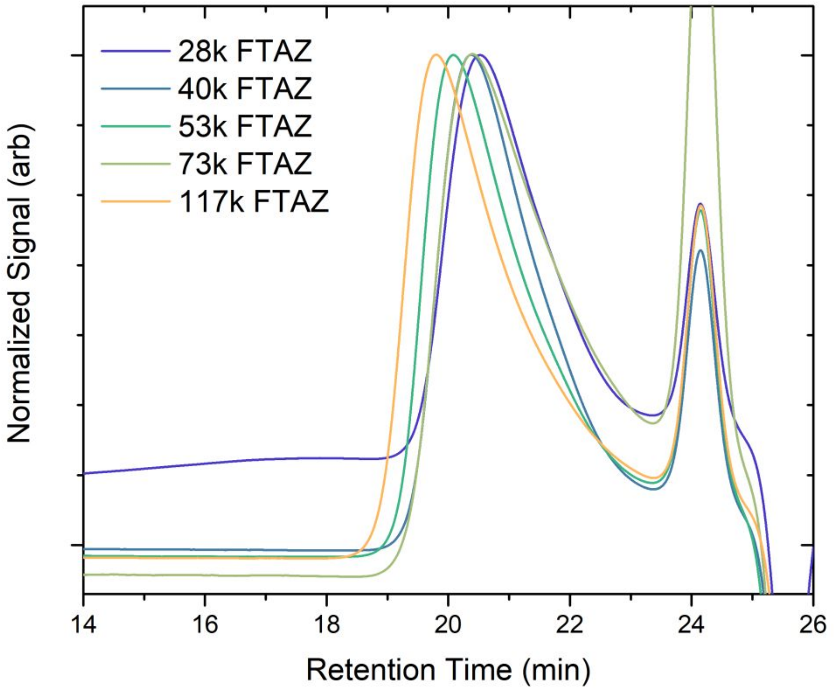


**Figure S1**. High Temperature Gel Permeation Chromatography (HT-GPC) traces of different batches of FTAZ

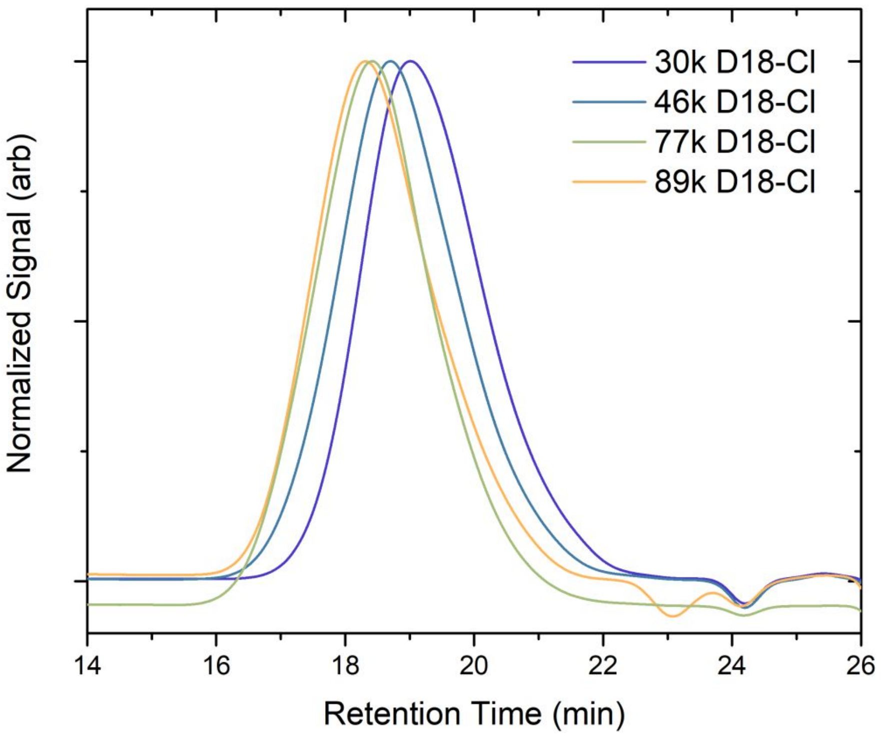


**Figure S2**. HT-GPC traces of different batches of D18-Cl

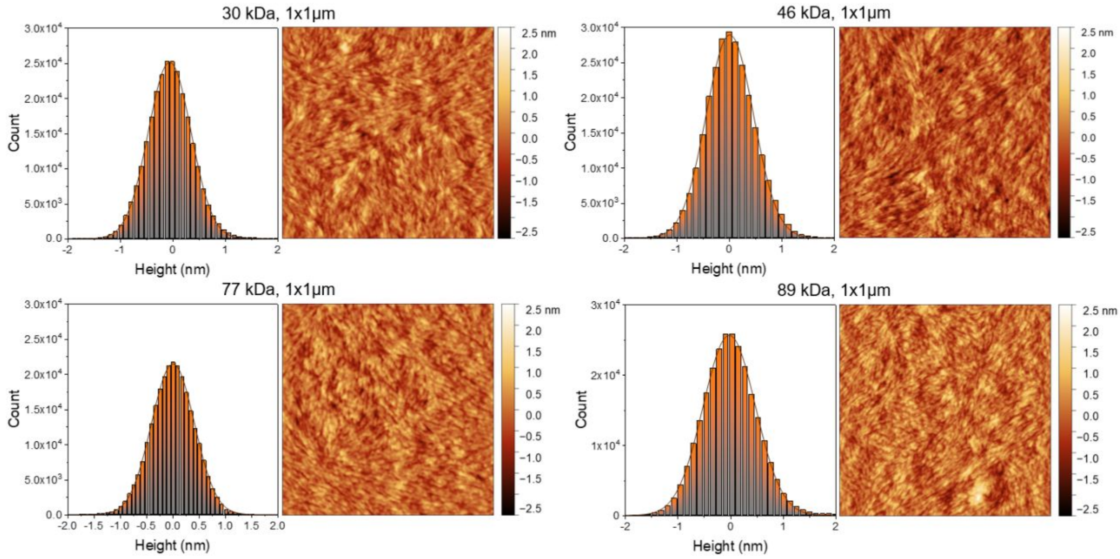


**Figure S3.** AFM topography images and height histogram of D18Cl samples.

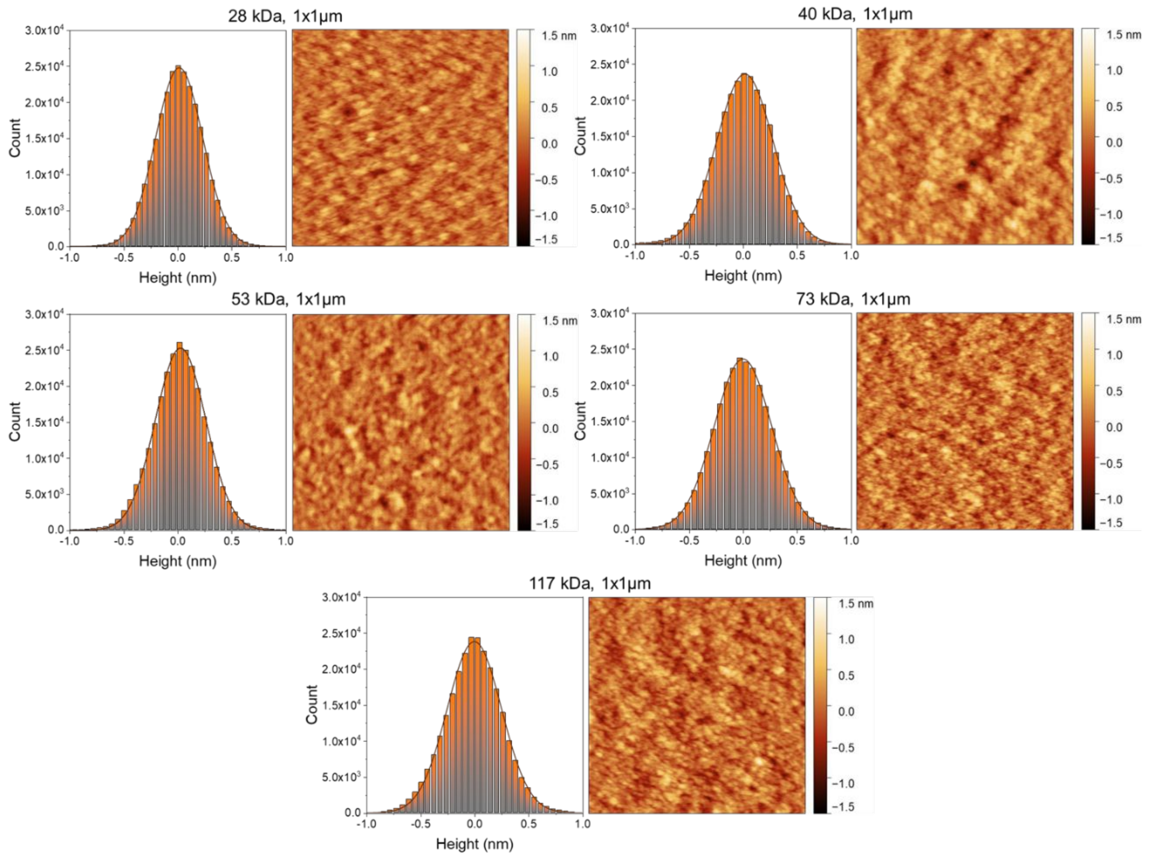


**Figure S4.** AFM topography images and height histogram of PBnDT-FTAZ samples.

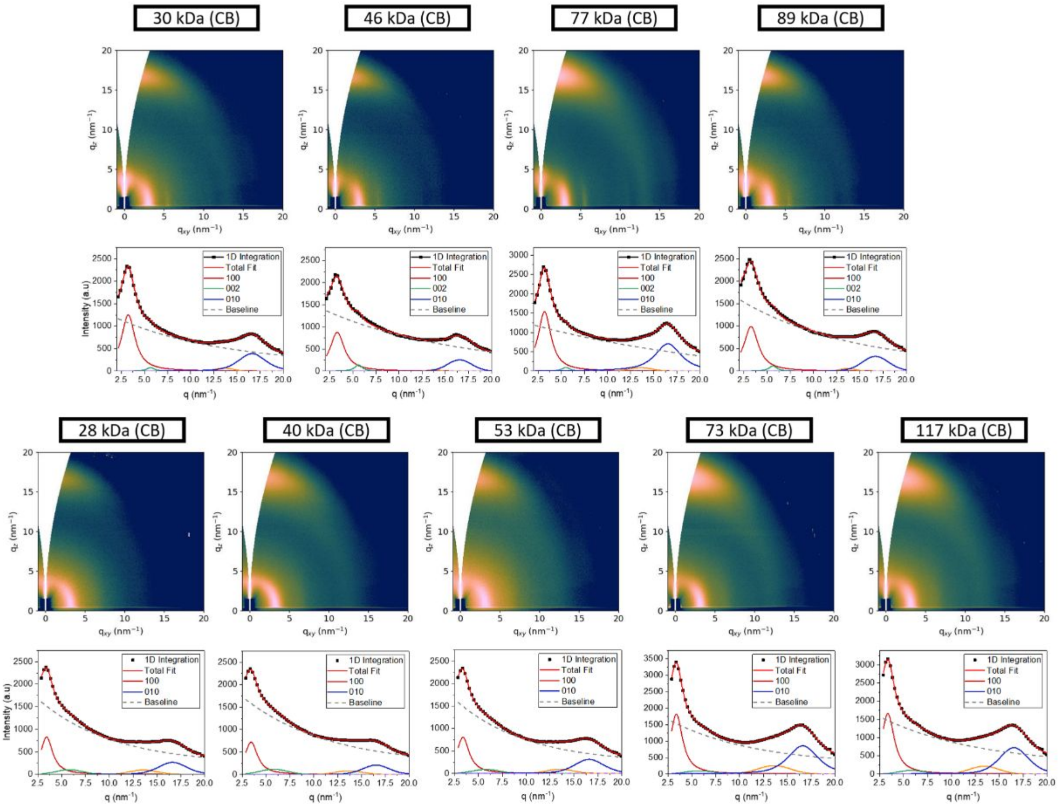


**Figure S5**. GIWAXS results obtained from as cast thin films of D18Cl and PBnDT-FTAZ: diffraction images and relative 1D integration cuts.

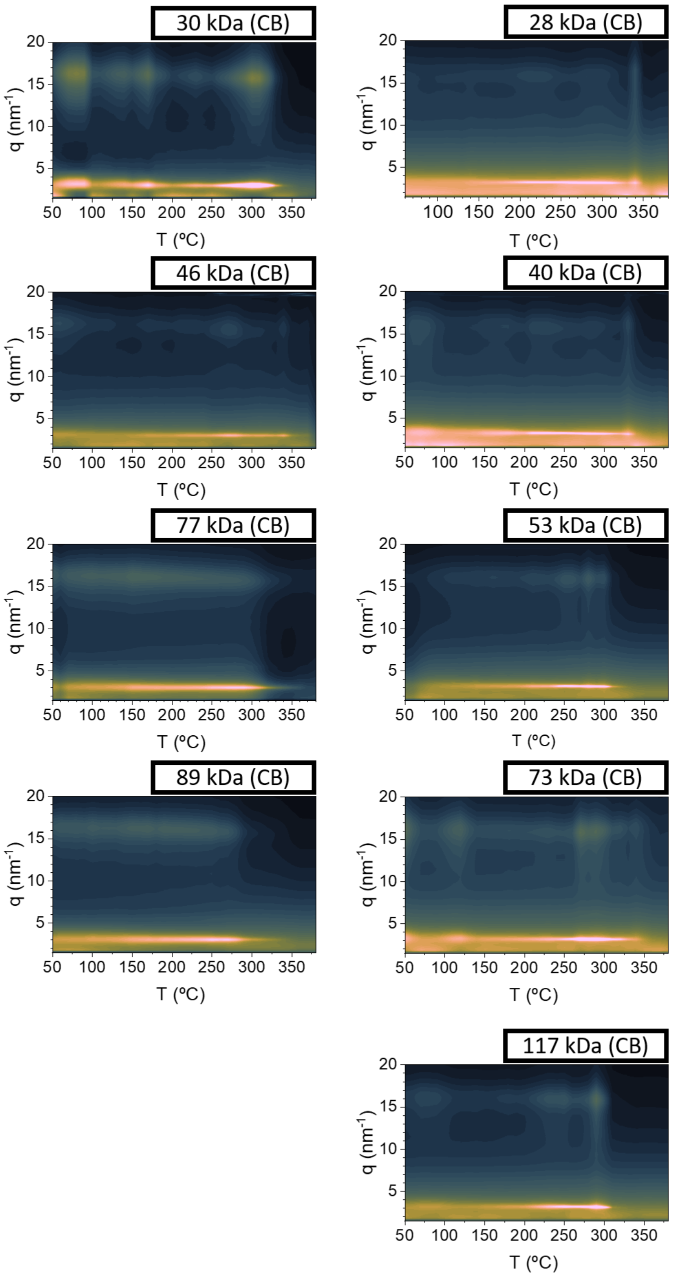


**Figure S6**. 1D integration of the GIWAXS patterns obtained from temperature-resolved in-situ experiment on D18Cl (77 kDa) and PBnDT-FTAZ (73 kDa).

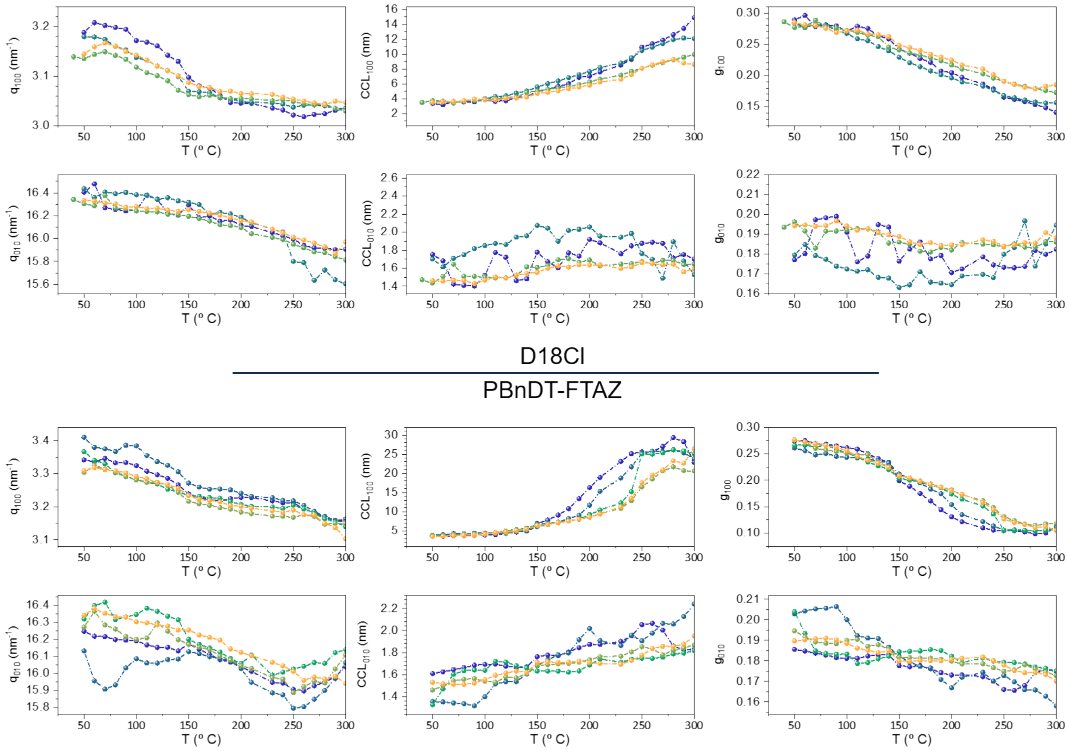


**Figure S7**. Structural parameters obtained from the GIWAXS diffractograms depicted in Figure S4.

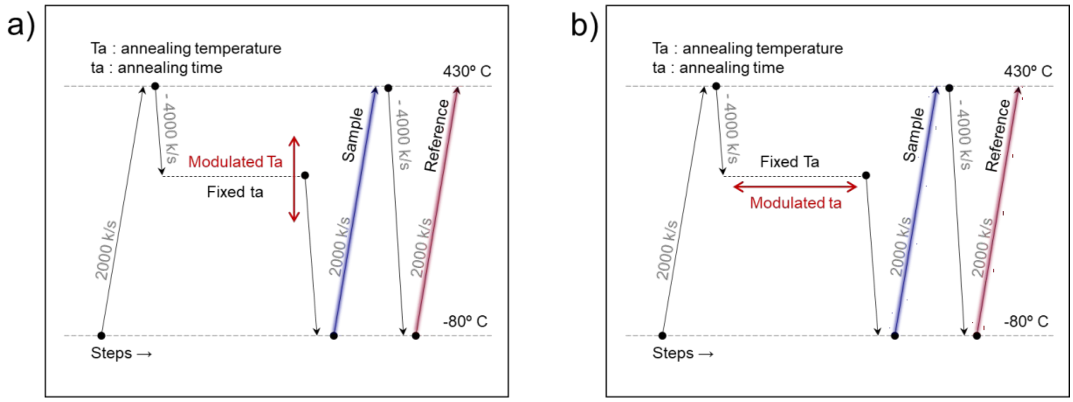


**Figure S8.** Representative schemes of the DSC protocols employed in this work.

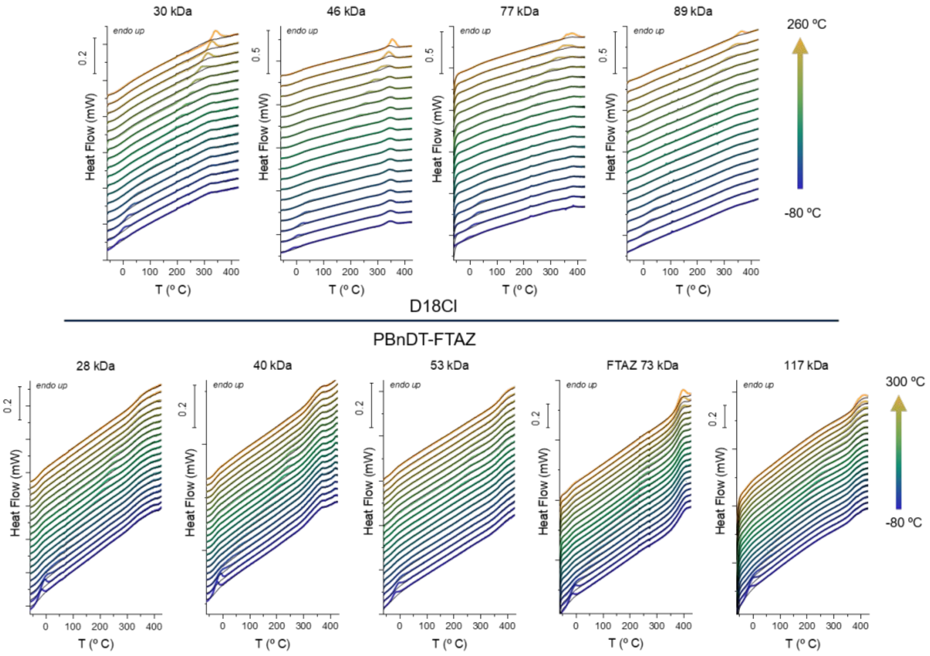


**Figure S9**. Thermograms representing the sample- and reference- lines obtained from FSC experiments following the protocol described in Figure S6a.

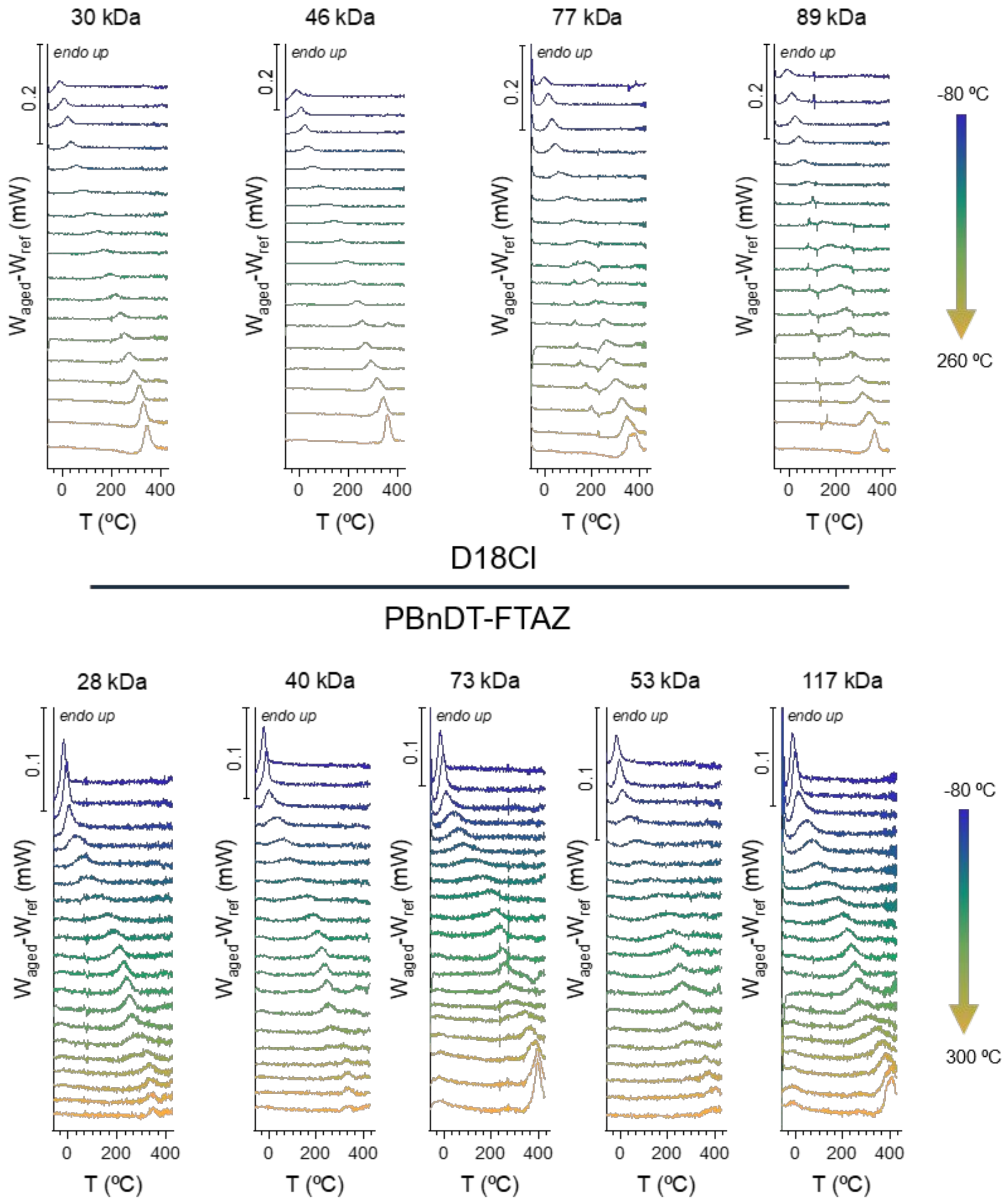


**Figure S10.** ΔHF curves (difference between sample-line and reference-line) obtained from data reported in Figure S9.

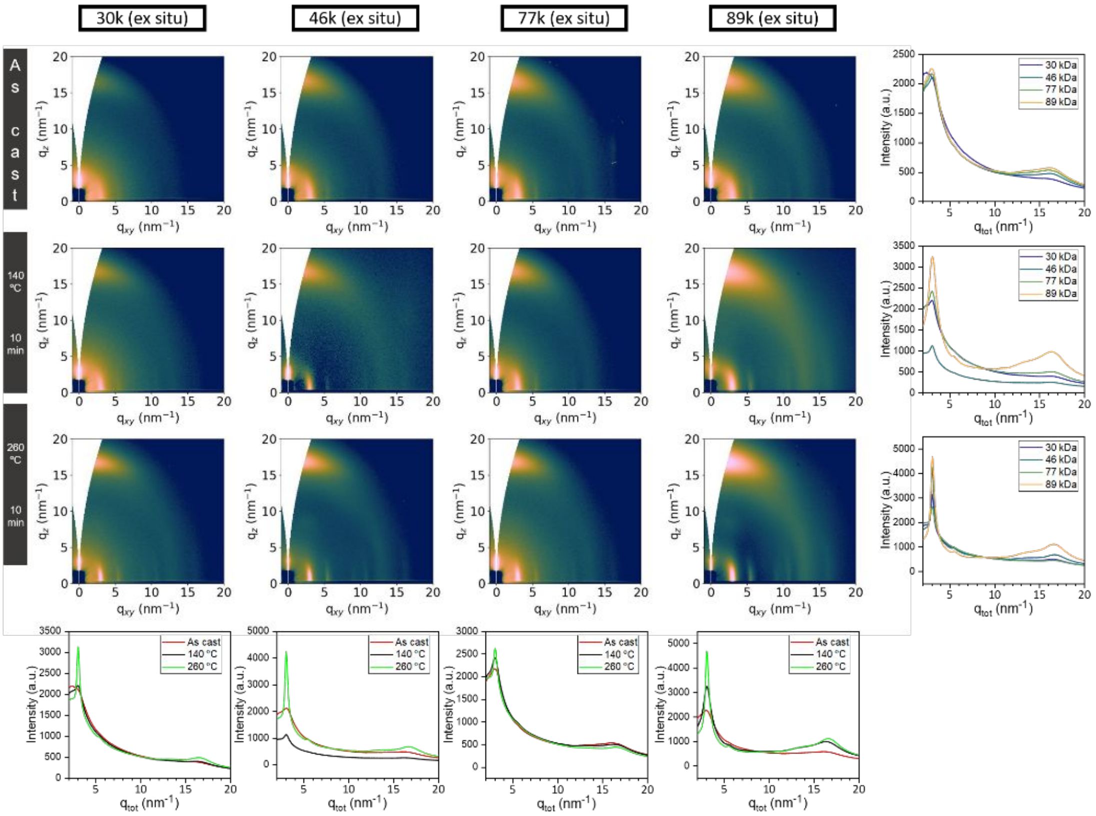


**Figure S11**. GIWAXS results obtained from D18Cl thin films under different conditions. From top to bottom, respectively: as cast, after 10 minutes annealing at 140 ºC, and after 10 minutes annealing at 260 ºC. On the right: 1D integration cuts of samples with same thermal history and different $M_n$. On the bottom: 1D integration cuts of samples with same $M_n$ and different thermal history.

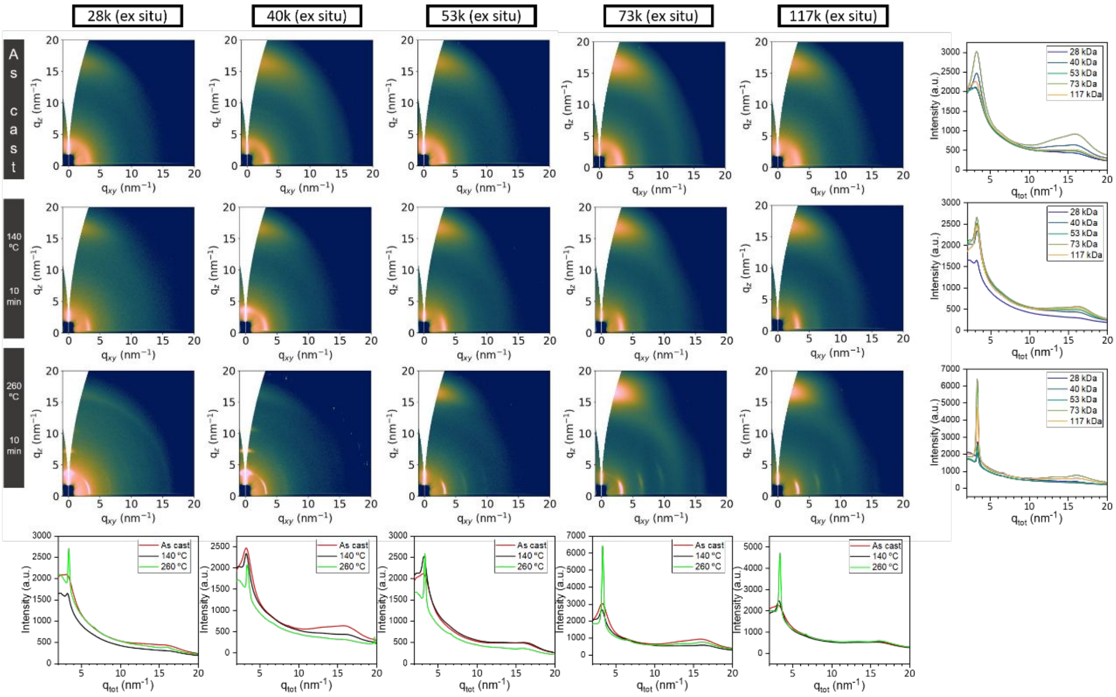

**Figure S12**. GIWAXS results obtained from PBnDT-FTAZ thin films under different conditions. From top to bottom, respectively: as cast, after 10 minutes annealing at 140 ºC, and after 10 minutes annealing at 260 ºC. On the right: 1D integration cuts of samples with same thermal history and different $M_n$. On the bottom: 1D integration cuts of samples with same $M_n$ and different thermal history.

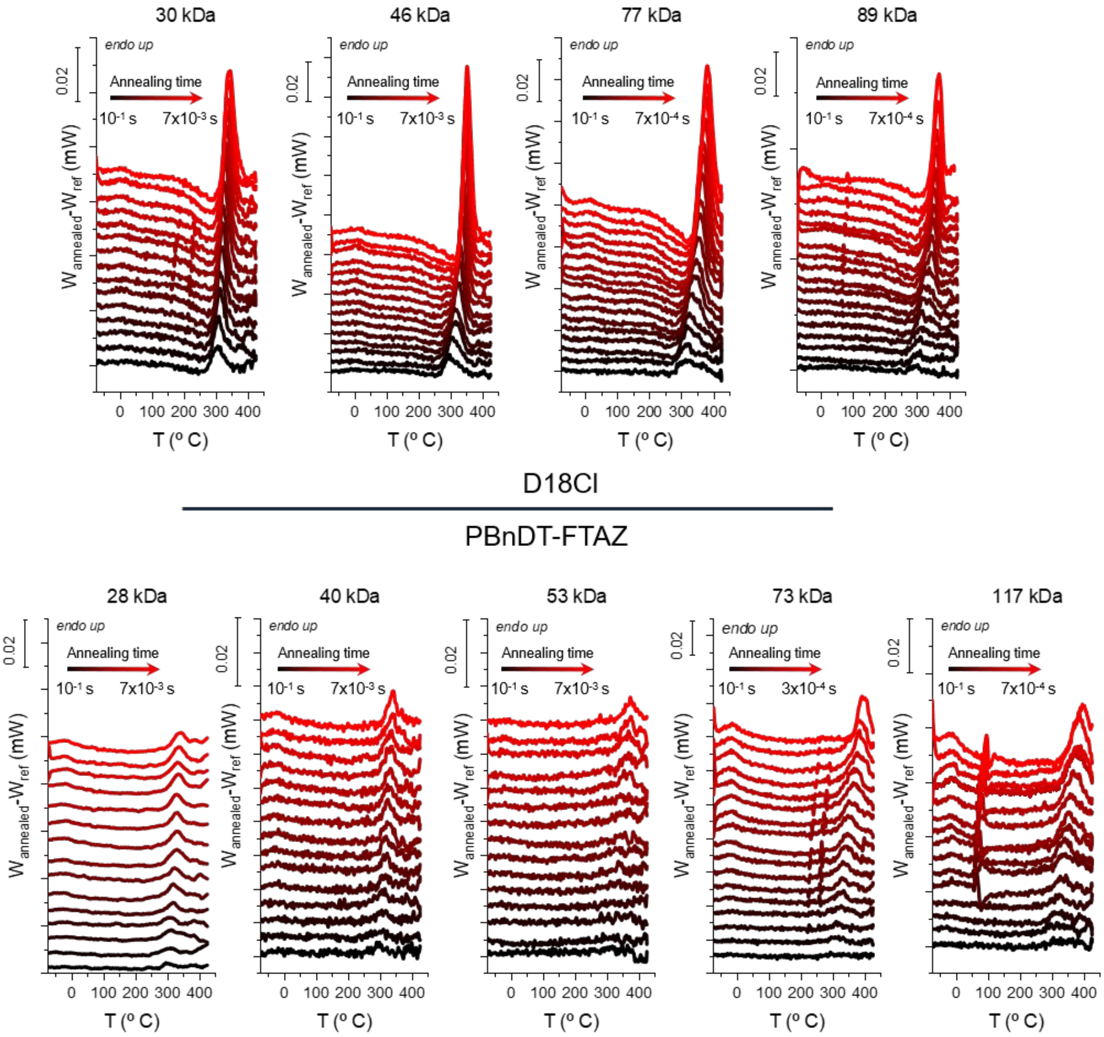


**Figure S13**. ΔHF curves (difference between sample-line and reference-line) obtained from FSC experiments performed following the experimental protocol resumed in Figure S8b, with $T_a$ fixed at 260 ºC.

## References

1. Price, S. C.; Stuart, A. C.; Yang, L.; Zhou, H.; You, W. Fluorine Substituted Conjugated Polymer of Medium Band Gap Yields 7% Efficiency in Polymer-Fullerene Solar Cells. *J. Am. Chem. Soc.* **2011**, *133* (12), 4625– 4631, DOI: 10.1021/ja1112595
